# Supplementary material for: Proteomic Studies of the Biofilm Matrix including Outer Membrane Vesicles of Burkholderia multivorans C1576, a Strain of Clinical Importance for Cystic Fibrosis
Source: Microorganisms. 2020 Nov 19;8(11):1826. doi: 10.3390/microorganisms8111826 (PMC7699398; doi:10.3390/microorganisms8111826)
Supplement: Supplementary file 1 [file microorganisms-08-01826-s001.pdf]

Table S1. Proteins identified in the biofilm matrix of *Burkholderia multivorans* C1576 after 7 days of grow at 30 °C.

Accession Number[a]	Description	Score[b]	# matches[c]	Gene name	Subcellular localization[d]	COG[e]	FIGFams[f]	MM (Da)	pI	Length (aa)
					Band 1 – Biofilm					
A0A1J9PJ01	"leucine ABC transporter subunit substrate-binding protein LivK [*Burkholderia catarinensis*]"	284	4	Bmul_3623	Periplasmic	E		38988.78	8.88	372
Q1BYB1	"Glutamate-1-semialdehyde 2,1-aminomutase OS=*Burkholderia cenocepacia* (strain AU 1054) OX=331271 GN=hemL PE=3 SV=1"	218	4	Bmul_2436	Cytoplasmic	H	FIG000338	44865.22	5.76	427
UPI00075D7ABF	"5-methyltetrahydropteroyltriglutamate--homocysteine S-methyltransferase [*Burkholderia multivorans*]"	212	8	*metE*	Cytoplasmic	E	FIG000683	85278.94	5.78	764
A4JJS1	"RecName: Full=5-methyltetrahydropteroyltriglutamate--homocysteine methyltransferase; AltName: Full=Cobalamin-independent methionine synthase; AltName: Full=Methionine synthase, vitamin-B12 independent isozyme [*Burkholderia vietnamiensis*] "	92	2	*metE*	Cytoplasmic	E	FIG000683	85939.77	5.88	767
J4R391	"transketolase [*Burkholderia multivorans*]"	182	5	*tktA*	Cytoplasmic	G	FIG020289	74368.32	5.87	690
A0A0H3KKQ4	"MULTISPECIES: pyruvate dehydrogenase (acetyl-transferring), homodimeric type [*Burkholderia*]"	179	3	*aceE*	Cytoplasmic	C	FIG138853	100784.1 7	5.61	898
A0A1B4MWH1	"MULTISPECIES: elongation factor G [*Burkholderia*]"	171	4	Bmul_0766	Cytoplasmic	J	FIG134254	77490.22	5.28	701
A0A0L1KUG7	"Translation elongation factor G [*Candidatus Burkholderia brachyanthoides*]"	121	3	Bmul_0246	Cytoplasmic	J	FIG134254	77613.56	5.23	700
A0A0H3KKE2	"phosphogluconate dehydrogenase (NADP(+)-dependent, decarboxylating) [*Burkholderia multivorans*]"	160	8	*gnd*	Cytoplasmic	G	FIG000405	51008.09	5.86	470
B9B4C9	"NADP-dependent isocitrate dehydrogenase [*Burkholderia multivorans*]"	153	7	Bmul_0771	Cytoplasmic	C	FIG001163	80243.56	5.79	741
A9ACU3	"Glycine dehydrogenase (decarboxylating) OS=*Burkholderia multivorans* (strain ATCC 17616 / 249) OX=395019 GN=gcvP PE=3 SV=1"	141	3	*gcvP*	Cytoplasmic	E	FIG000700	104328.6 1	5.99	975
UPI000277C7B3	"ribosomal protein S1 [*Burkholderia multivorans* ATCC BAA-247]"	140	9	*rpsA*	Cytoplasmic	J	FIG000210	65159.90	5.20	596
UPI00086385B4	"4-hydroxyphenylpyruvate dioxygenase [*Burkholderia contaminans*]"	140	3	Bmul_0223	Cytoplasmic	E R		41144.51	5.61	365
Q5NSX4	"MULTISPECIES: DNA polymerase III subunit beta [*Burkholderia*]"	137	3	*dnaN*	Cytoplasmic	L	FIG066425	40699.87	5.42	368
B9BGB9	"peptidase S1 [*Burkholderia multivorans*]"	137	2	Bmul_3442	Extracellular	E	FIG006033	60428.61	5.87	558
UPI0005367B45	"transcription termination factor NusA [*Burkholderia pseudomallei* MSHR4303]"	130	2	*nusA*	Cytoplasmic	K	FIG000168	54119.40	4.59	485

B9B3T5	chaperone SurA (Peptidyl-prolyl cis-trans isomerase surA) (PPIase surA) (Rotamase surA) [*Burkholderia multivorans* CGD1]"	126	2	*surA*	Periplasmic	O	FIG036406	49346.62	7.84	453
A0A1B4TUZ2	"MULTISPECIES: chaperonin GroEL [*Burkholderia*]"	120	3	Bmul_2528	Cytoplasmic	O	FIG000056	57089.49	5.13	546
UPI0006C7D81D	"aldehyde dehydrogenase family protein [*Burkholderia multivorans*]"	119	4	Bmul_3451	Cytoplasmic	C		53822.70	6.11	503
UPI00066861A4	"aconitate hydratase AcnA, partial [*Burkholderia multivorans*]"	116	2	Bmul_4655	Cytoplasmic	C	FIG138200	72387.94	6.09	662
UPI0006C79FF8	"MULTISPECIES: porin [*Burkholderia*]"	116	3	*opcP*	Outer Membrane	M	FIG005925	38840.78	9.44	373
Q1BRB0	"ATP synthase subunit beta OS=*Burkholderia cenocepacia* (strain AU 1054) OX=331271 GN=atpD PE=3 SV=1"	109	2	*atpD*	Cytoplasmic	C	FIG040241	50582.99	5.25	464
Q1BYX3	"Chaperone protein DnaK OS=*Burkholderia cenocepacia* (strain AU 1054) OX=331271 GN=dnaK PE=3 SV=1"	108	2	*dnaK*	Cytoplasmic	O	FIG134874	69830.16	4.90	650
A0A0H3KHD1	"MULTISPECIES: NADP-dependent isocitrate dehydrogenase [*Burkholderia*]"	107	2	Bmul_0770	Cytoplasmic	C	FIG000600	45879.75	5.32	418
UPI00015FD920	"Catalase [*Burkholderia multivorans* ATCC 17616]"	104	2	*katE*	Cytoplasmic	P	FIG000313	79858.52	6.26	728
UPI0002F1A962	"malate synthase family protein [*Burkholderia mallei*]"	102	1	Bmul_1225	Cytoplasmic	C	FIG001217	40878.60	5.57	367
A0A0H3KJ85	"dihydrolipoamide dehydrogenase [*Burkholderia stabilis*]"	95	2	Bmul_1745	Cytoplasmic	C	FIG013010	50549.17	5.90	476
UPI0003C75E9F	"leucine ABC transporter substrate-binding protein [*Pandoraea pnomenusa* 3kgm]"	87	1	Bmul_3623	Periplasmic	E		39027.75	8.98	370
UPI000B9248D3	fumarylacetoacetase, partial [*Burkholderia multivorans*]			*fah*	Cytoplasmic	Q	FIG001596	42480.20	5.86	393
A0A1W0ZLF4	"class I SAM-dependent methyltransferase [*Burkholderia multivorans*]"	83	1	Bmul_0727	Cytoplasmic	M	FIG001555	46095.40	6.06	406
Q0BAV4	"Glutamyl-tRNA(Gln) amidotransferase subunit A OS=*Burkholderia ambifaria* (strain ATCC BAA-244 / AMMD) OX=339670 GN=gatA PE=3 SV=1"	82	1	Bmul_3105	Cytoplasmic	J	FIG000359	52443.25	5.88	496
E5AQT2	"transcription termination/antitermination protein NusA [*Paraburkholderia rhizoxinica*]"	81	2	*nusA*	Cytoplasmic	K	FIG000168	55257.69	4.60	491
B5WJ78	"isocitrate lyase [*Burkholderia* sp. H160]"	80	1	*aceA*	Cytoplasmic	C	FIG000818	48052.20	5.55	436
A0A1J9PMN7	"citrate (Si)-synthase [*Burkholderia catarinensis*]"	80	3	Bmul_4643	Cytoplasmic	C	FIG138183	48464.70	6.32	433
A0A1J9NQ05	"AMP-dependent synthetase [*Burkholderia catarinensis*]"	80	2	Bmul_3657	Cytoplasmic	I	FIG022631	63925.94	5.86	569
Q62G22	"Adenosylhomocysteinase OS=*Burkholderia mallei* (strain ATCC 23344) OX=243160 GN=ahcY PE=3 SV=1"	78	1	*ahcY*	Cytoplasmic	H	FIG000858	52201.28	5.73	473
A2S989	"phosphoglucomutase/phosphomannomutase family protein [*Burkholderia mallei* NCTC 10229]"	76	1	Bmul_2497	Cytoplasmic	G		50111.94	5.23	464
A0A0L1KUV9	"NADP-specific glutamate dehydrogenase [*Candidatus Burkholderia brachyanthoides*]"	73	1	Bmul_2715	Cytoplasmic	E	FIG137408	47282.75	6.12	437
Biofilm - Band 2										

B9AYL8	"MULTISPECIES: branched-chain amino acid ABC transporter substrate-binding protein [*Burkholderia*]"	723	16	Bmul_3623<br>*livK*	Periplasmic	E		39044.85	8.88	372
UPI0008639508	"beta-ketoacyl-ACP reductase [*Burkholderia contaminans*]"	587	17	Bmul_2181	Cytoplasmic	I<br>Q<br>R	FIG004337	25639.38	7.86	249
B9B7H9	"malate dehydrogenase [*Burkholderia multivorans*]"	470	13	*mdh*	Unknown	C	FIG000455	35174.43	5.91	328
A0A2S9MBH9	"iron ABC transporter substrate-binding protein [*Burkholderia multivorans*]"	313	11	Bmul_2093	Periplasmic	P	FIG147656	37459.93	8.97	347
A0A1B4MZB5	"MULTISPECIES: ABC transporter substrate-binding protein [*Burkholderia*]"	312	13	Bmul_3347	Periplasmic	E<br>T	FIG084786	28144.24	8.82	260
A0A0H3KKM9	"MULTISPECIES: amino acid ABC transporter substrate-binding protein [*Burkholderia*]"	255	10	Bmul_2714	Periplasmic	E<br>T		32657.40	9.12	297
A0A0H3KIM0	"branched-chain amino acid ABC transporter substrate-binding protein [*Burkholderia multivorans*]"	169	4	Bmul_0115	Periplasmic	E	FIG006112	40150.04	8.90	379
J4RH02	"hypothetical protein [*Burkholderia multivorans*]" purine nucleoside permease [*Burkholderia multivorans*]	153	3	Bmul_3369	Unknown	F	FIG020819	38730.47	6.74	357
A0A2N9D2H7	"Cystine-binding periplasmic protein [*Burkholderia cenocepacia*]"	139	3	Bmul_0351<br>*fliY*	Periplasmic	E<br>T	FIG019050	28844.14	9.33	266
UPI000277CBCE	"ABC transporter, solute-binding protein [*Burkholderia multivorans* ATCC BAA-247]"	130	4	Bmul_1509	Periplasmic	E	FIG143081	39662.55	8.76	360
U2G3Y5	"Putrescine ABC transporter putrescine-binding protein PotF [*Burkholderia* sp. AU4i]"	81	2	Bmul_1509	Periplasmic	E	FIG143081	39475.29	8.62	359
A0A1J9PBM8	"ABC transporter substrate-binding protein [*Burkholderia catarinensis*]"	125	4	Bmul_0472	Unknown	R	FIG003627	33206.33	9.60	321
B9B9K6	"2-(1,2-epoxy-1,2-dihydrophenyl)acetyl-CoA isomerase [*Burkholderia multivorans*]"	123	1	Bmul_2853<br>*paaB*	Cytoplasmic	I	FIG138179	28243.15	5.62	263
UPI00015FD976	"N-acetyl-gamma-glutamyl-phosphate reductase [*Burkholderia multivorans* ATCC 17616]"	122	1	Bmul_0217	Cytoplasmic	E	FIG000358	35962.73	6.34	331
G4MAY8	"Phenylacetate degradation enoyl-CoA hydratase PaaA [*Candidatus Paraburkholderia kirkii* UZHbot1]"	120	1	Bmul_2850<br>*paaA*	Cytoplasmic	I	FIG003653	27738.13	5.77	258
Q8RL37	"carboxyvinyl-carboxyphosphonate phosphorylmutase, partial [*Burkholderia multivorans*]"	115	2	*prpB*	Cytoplasmic	G	FIG003486	17318.76	5.43	159
UPI000863A009	"electron transfer flavoprotein subunit beta [*Burkholderia contaminans*]"	112	3	Bmul_2241	Unknown	C	FIG000366	31990.31	4.84	315
UPI000D337244	"alcohol dehydrogenase [*Burkholderia multivorans*]"	110	2	Bmul_5300	Unknown	C	FIG022830	34107.56	5.42	315
Q1BHJ3	"2,3,4,5-tetrahydropyridine-2,6-dicarboxylate N-succinyltransferase OS=*Burkholderia cenocepacia* (strain AU 1054) OX=331271 GN=*dapD* PE=3 SV=1"	110	2	*dapD*	Cytoplasmic	E	FIG000908	29479.56	5.66	275
A1V8S5	"Uroporphyrinogen decarboxylase OS=*Burkholderia mallei* (strain SAVP1) OX=320388 GN=hemE PE=3 SV=1"	106	1	*hemE*	Cytoplasmic	H	FIG000428	39457.82	5.75	364
F0G8P7	"carboxymethylenebutenolidase [*Burkholderia* sp. TJI49]"	103	1	Bmul_0184	Unknown	Q	FIG004936	24462.57	5.95	230
A2W9A3	"Translation elongation factor Ts [*Burkholderia dolosa*	96	4	*tsf*	Cytoplasmic	J	FIG000191	31325.03	5.38	294

	AU0158]"									
B9B504	"quinone oxidoreductase [*Burkholderia multivorans*]"	97	1	Bmul_0988 *qor*	Cytoplasmic	C R	FIG005137	34406.33	6.32	324
UPI000B91FAA6	"acetoin dehydrogenase dihydrolipoyllysine-residue acetyltransferase subunit, partial [*Burkholderia multivorans*]"	96	1	Bmul_1436	Unknown	C R	FIG006936	28863.77	5.79	274
UPI0006678661	"branched chain amino acid ABC transporter substrate-binding protein, partial [*Burkholderia cenocepacia*]"	94	2	Bmul_0186	Periplasmic	E	FIG007505	24622.93	6.51	238
B9BM23	"alcohol dehydrogenase, zinc-binding domain protein [*Burkholderia multivorans* CGD2]"	90	1	Bmul_1494	Cytoplasmic	C R	FIG000618	37996.62	5.82	355
B9AYT4	"C4-dicarboxylate ABC transporter [*Burkholderia multivorans*]"	80	3	Bmul_3681	Periplasmic	G	FIG016663	36761.13	9.36	331
F0G773	"phosphate ABC transporter, periplasmic phosphate-binding protein [*Burkholderia* sp. TJI49]"	80	1	Bmul_2020 PstS	Periplasmic	P	FIG010508	36131.24	9.01	343
A0A1J9NUV0	"ABC transporter [*Burkholderia catarinensis*]"	79	1	Bmul_3513	Cytoplasmic Membrane	M		32085.86	8.99	307
A0A1J9PYN8	"DUF1338 domain-containing protein [*Burkholderia catarinensis*]"	76	1	Bmul_5159	Unknown	S? EGNOGG	FIG140295	38346.22	5.69	342
B9B483	"thioredoxin [*Burkholderia multivorans*]"	72	1	*ybbN*	Cytoplasmic	O	FIG000875	31382.52	4.64	282
UPI0007567985	"electron transfer flavoprotein subunit alpha [*Burkholderia ubonensis*]"	70	2	Bmul_2241	Unknown	C	FIG000366	31972.38	4.76	314
A0A084DKJ7	"electron transporter RnfB, partial [*Burkholderia* sp. MSh2]"	69	1	Bmul_2242	Cytoplasmic	C	FIG136866	24153.01	5.44	228
UPI0000E9391B	"phosphoglycerate mutase 1 family [*Burkholderia multivorans* ATCC 17616]"	69	1	*gpmA*	Cytoplasmic	G	FIG006157	30168.41	7.80	270
	Biofilm – Band 3									
A0A1B4MR97	"MULTISPECIES: thiol peroxidase [*Burkholderia*]"	709	30	*tpx*	Periplasmic	O	FIG002857	17386.78	5.33	167
A0A3P0MVL3	"thiol peroxidase [*Burkholderia ambifaria*]"	239	7	*tpx*	Periplasmic	O	FIG002857	17197.57	5.30	167
A0A1B4BPF2	"thiol peroxidase [*Burkholderia diffusa*]"	213	8	*tpx*	Periplasmic	O	FIG002857	17255.65	5.33	167
F0G9I6	"carboxymuconolactone decarboxylase [*Burkholderia* sp. TJI49]"	526	22	*ahpD*	Cytoplasmic	S	FIG003720	18832.53	5.80	175
B9B376	"MULTISPECIES: thiol:disulfide interchange protein DsbA/DsbL [*Burkholderia*]"	350	15	*dsbA*	Periplasmic	OC	FIG133105	23276.92	9.05	212
UPI000055A384	"redoxin family protein [*Burkholderia mallei*]"	179	7	Bmul_1331	Cytoplasmic	O	FIG000710	18916.31	5.02	169
A0A0H3KH36	"MULTISPECIES: superoxide dismutase [*Burkholderia*]"	173	6	*sod*	Periplasmic	P	FIG133139	21100.63	5.62	192
UPI000B920051	"riboflavin synthase [*Burkholderia multivorans*]"	169	6	*ribE*	Cytoplasmic	H	FIG000273	22069.93	4.96	209
UPI0000E94711	"hypothetical protein UA17_02969 [*Burkholderia multivorans*]"	156	8	Bmul_2263	Unknown	S	FIG005158	21445.71	9.36	200
UPI0008639508	"beta-ketoacyl-ACP reductase [*Burkholderia contaminans*]"	151	1	Bmul_2181	Cytoplasmic	IQR	FIG004337	25639.38	7.86	249
B9BCY0	"YceI family protein [*Burkholderia multivorans* CGD1]"	136	5	Bmul_2669	Unknown	S	FIG005976	20383.08	6.29	188

F0G8P7	"carboxymethylenebutenolidase [*Burkholderia* sp. TJI49]"	132	1	Bmul_0184	Unknown	Q	FIG004936	24462.57	5.95	230
A0A0H3KFZ4	"MULTISPECIES: peptidyl-prolyl cis-trans isomerase [*Burkholderia*]"	127	6	*ppiB*	Cytoplasmic	O	FIG000999	17822.04	5.93	163
A0A1J9NTF1	"peroxidase [*Burkholderia catarinensis*]"	125	4	Bmul_2571	Cytoplasmic	O	FIG133247	23793.05	5.61	212
ACP_BURCA	"Acyl carrier protein OS=*Burkholderia cenocepacia* (strain AU 1054) OX=331271 GN=acpP PE=3 SV=1"	125	3	*acpP*	Cytoplasmic	IQ	FIG002328	8711.74	4.08	79
B9BEK8	"ATP-dependent Clp protease, proteolytic subunit ClpP [*Burkholderia multivorans* CGD1]"	121	3	Bmul_1349 *clpP*	Cytoplasmic	OU	FIG000028	22730.02	5.43	208
UPI0007567985	"electron transfer flavoprotein subunit alpha [*Burkholderia ubonensis*]"	118	4	Bmul_2241	Unknown	C	FIG000366	31972.38	4.76	314
J4RD71	"MULTISPECIES: ABC transporter substrate-binding protein [*Burkholderia*]"	117	3	Bmul_3185 *ttg2D*	Unknown	Q	FIG002330	21462.31	9.62	198
B9B9H6	"bifunctional glutamate N-acetyltransferase/amino-acid acetyltransferase ArgJ [*Burkholderia multivorans*]"	116	3	*argJ*	Cytoplasmic	E	FIG000630	43333.38	5.26	413
B9BQJ9	"ecotin [*Burkholderia multivorans*]"	113	2	*ecoT*	Periplasmic	R	FIG019473	18081.74	8.38	165
SECB_BURCA	"Protein-export protein SecB OS=*Burkholderia cenocepacia* (strain AU 1054) OX=331271 GN=secB PE=3 SV=1"	107	8	*secB*	Cytoplasmic	U	FIG001903	17897.39	4.49	164
W5U255	"poly(R)-hydroxyalkanoic acid synthase, partial [*Paraburkholderia caledonica*]"	107	2	Bmul_1485 *phaC*	Cytoplasmic	IQR	FIG055441	16454.53	6.10	151
UPI0001821B56	"Inorganic diphosphatase [*Burkholderia* sp. H160]"	105	1	*ppa*	Cytoplasmic	C	FIG000429	19280.18	5.32	175
A0A238H9F9	"Single-stranded DNA-binding protein [*Burkholderia singularis*]"	102	2	Bmul_0538	Cytoplasmic	L	FIG107367	20480.75	6.75	194
UPI0005104257	"electron transfer flavoprotein subunit alpha [*Burkholderia multivorans*]"	101	3	Bmul_2241	Unknown	C	FIG000366	31584.90	4.83	310
UPI00050DFB22	"phosphohistidine phosphatase SixA [*Burkholderia multivorans*]"	98	2	*sixA*	Unknown	T	FIG001992	16410.62	6.09	152
A9AJQ9	"Trigger factor OS=*Burkholderia multivorans* (strain ATCC 17616 / 249) OX=395019 GN=tig PE=3 SV=1"	97	1	*tig*	Cytoplasmic	O	FIG000286	49750.27	5.01	448
Q39JG2	"MULTISPECIES: YceI family protein [*Burkholderia*]"	91	2	Bmul_2670	Periplasmic	S	FIG002558	19582.32	9.32	186
RRF_BURCJ	"Ribosome-recycling factor OS=*Burkholderia cenocepacia* (strain ATCC BAA-245 / DSM 16553 / LMG 16656 / NCTC 13227 / J2315 / CF5610) OX=216591 GN=frr PE=3 SV=1"	87	2	*frr*	Cytoplasmic	J	FIG000202	20812.91	7.88	186
UPI00018231F2	"peptidyl-prolyl cis-trans isomerase cyclophilin type [*Burkholderia* sp. H160]"	82	2	Bmul_1201	Periplasmic	O		20480.37	9.33	193
B1FF14	"toluene tolerance family protein [*Burkholderia ambifaria* IOP40-10]"	81	1	Bmul_0321	Unknown	Q	FIG002330	23791.20	9.49	210
A4JBR9 (GRPE_BURVG)	"Protein GrpE OS=*Burkholderia vietnamiensis* (strain G4 / LMG 22486) OX=269482 GN=grpE PE=3 SV=1"	78	1	*grpE*	Cytoplasmic	O	FIG000103	19313.72	5.05	181
UPI00075AF589	"hypothetical protein WJ29_09985 [*Burkholderia ubonensis*]"	76	1	Bmul_0927	Unknown	J		16099.45	5.67	155
A0A381BPK5	"carboxymuconolactone decarboxylase family protein	71	2	*ahpD*	Cytoplasmic	S	FIG003720	18627.31	5.92	175

	[*Burkholderia oklahomensis*]"									
					Biofilm – Band 4					
Q1BXV1 (ACP_BURCA)	"Acyl carrier protein OS=*Burkholderia cenocepacia* (strain AU 1054) OX=331271 GN=acpP PE=3 SV=1"	389	15	*acpP*	Cytoplasmic	IQ	FIG002328	8711.74	4.08	79
Q2SWG0	"thioredoxin [*Burkholderia thailandensis* E264]"	275	9	Bmul_1445	Cytoplasmic	OC		15721.97	8.46	146
J5B7D3	"MULTISPECIES: transporter [*Burkholderia*]"	162	3	Bmul_6152	Cytoplasmic	R	FIG035112	14093.11	7.91	141
A2WC84	"Chaperonin Cpn10 [*Burkholderia dolosa* AU0158]"	155	4	Bmul_2529 *groS*	Cytoplasmic	O	FIG138328	10721.41	5.79	99
UPI00041BF7D8	"co-chaperone GroES [*Paraburkholderia mimosarum*]"	88	3	Bmul_2529	Cytoplasmic	O	FIG138328	10423.05	5.79	96
J4RCD6	"MULTISPECIES: RidA family protein [*Burkholderia*]"	150	7	Bmul_0927	Unknown	J		15740.07	5.91	152
ABX16815 B9BDG5	"thioesterase superfamily protein [*Burkholderia multivorans* ATCC 17616]"	129	2	Bmul_3131	Cytoplasmic	Q	FIG005542	16591.14	6.83	153
UPI0005534760	"PTS sugar transporter subunit IIA [*Burkholderia multivorans*]"	123	4	*ptsN*	Cytoplasmic	GT	FIG003918	18799.49	6.30	170
A0A1W0YJS2	"HIT family protein [*Burkholderia multivorans*]"	120	3	Bmul_3327	Cytoplasmic	FGR	FIG009257	14772.95	5.79	139
A0A1J9PMB8	"histidine triad nucleotide-binding protein [*Burkholderia catarinensis*]"	118	3	Bmul_0338	Cytoplasmic	FGR	FIG146285	13046.95	6.35	121
A0A1J9PIU8	"cytochrome C [*Burkholderia catarinensis*]"	103	1	Bmul_0946	Periplasmic	C	FIG143085	12672.44	9.08	119
A9AJW4 (RL9_BURM1)	"50S ribosomal protein L9 OS=*Burkholderia multivorans* (strain ATCC 17616 / 249) OX=395019 GN=rplI PE=3 SV=1"	102	1	*rplL*	Cytoplasmic	J	FIG000205	16274.76	6.85	150
WP_006414074.1	"DUF2147 domain-containing protein [*Burkholderia multivorans*]"	99	2	Bmul_1742	Periplasmic	S	FIG005742	16546.98	8.36	153
A9ADI4 (RL7_BURM1)	"50S ribosomal protein L7/L12 OS=*Burkholderia multivorans* (strain ATCC 17616 / 249) OX=395019 GN=rplL PE=3 SV=1"	99	5	*rplL*	Unknown	J	FIG000141	12529.47	4.90	124
UPI0007585CF4	"transcription elongation factor GreAB [*Burkholderia territorii*]"	96	2	*rnk*	Cytoplasmic	K	FIG001255	14776.00	5.35	132
UPI000B91E037	"transcriptional regulator, partial [*Burkholderia multivorans*]"	86	1	Bmul_2098	Unknown	K	FIG001194	8556.72	4.91	79
A0A1B4N3W2	"MULTISPECIES: CsbD family protein [*Burkholderia*]"	84	1	Bmul_5157 *csbD*	Unknown	S		7568.51	5.37	65
A2W724	"hypothetical protein BDAG_00461 [*Burkholderia dolosa* AU0158]"	84	2	Bmul_0386	Unknown	J	FIG006243	18085.49	6.29	167
A0A1J9NH02	"glyoxalase [*Burkholderia catarinensis*]"	83	1	Bmul_2750	Unknown	S	FIG005966	15463.42	4.74	138
Q1BNU0 (PPNP_BURCA)	"Pyrimidine/purine nucleoside phosphorylase OS=*Burkholderia cenocepacia* (strain AU 1054) OX=331271 GN=ppnP PE=3 SV=1"	82	2	*ppnP*	Unknown	S	FIG019036	11608.18	4.99	106
A1TM32 (HFQ_ACIAC)	"RNA-binding protein Hfq OS=*Acidovorax citrulli* (strain AAC00-1) OX=397945 GN=hfq PE=3 SV=1"	81	2	Bmul_1468 *hfq*	Cytoplasmic	R	FIG000583	9200.50	6.71	83
B9B285	"OsmC family protein [*Burkholderia multivorans* CGD1]"	81	1	Bmul_4937	Cytoplasmic	O	FIG005718	20566.84	10.5 4	183

A0A1J9PT95	"hypothetical protein BFF94_26245 [*Burkholderia catarinensis*]"	80	1	Bmul_2928	Extracellular	S	FIG009175	18367.54	6.65	167
Q1BHA4 (ISPF_BURCA)	"2-C-methyl-D-erythritol 2,4-cyclodiphosphate synthase OS=*Burkholderia cenocepacia* (strain AU 1054) OX=331271 GN=ispF PE=3 SV=1"	80	1	*ispF*	Cytoplasmic	I	FIG137327	16923.36	6.19	161
A0A0H3KRN6	"MULTISPECIES: DUF493 domain-containing protein [*Burkholderia*]"	79	2	Bmul_0413	Cytoplasmic	S	FIG002301	11318.94	5.12	102
B4EEZ0 (ATPE_BURCJ)	"ATP synthase epsilon chain OS=*Burkholderia cenocepacia* (strain ATCC BAA-245 / DSM 16553 / LMG 16656 / NCTC 13227 / J2315 / CF5610) OX=216591 GN=atpC PE=3 SV=1"	79	1	*atpC*	Cytoplasmic	C	FIG000249	15013.16	5.11	141
A2W7T2	"hypothetical protein BDAG_00728 [*Burkholderia dolosa* AU0158]"	73	2	Bmul_0683	Unknown	S EGNOGG	FIG004968	16330.25	8.76	155
UPI000B923569	"hypothetical protein CA831_04455, partial [*Burkholderia multivorans*]"	70	1	Bmul_4929	Unknown	S	FIG005605	12776.12	5.69	117
A0A238H8X3	"Succinyl-CoA ligase [ADP-forming] alpha chain [*Burkholderia singularis*]"	70	1	*sucD*	Cytoplasmic	C	FIG038611	32631.76	8.96	309
A9AJG1 (ATPD_BURM1)	"ATP synthase subunit delta OS=*Burkholderia multivorans* (strain ATCC 17616 / 249) OX=395019 GN=atpH PE=3 SV=1"	70	1	*atpH*	Cytoplasmic	C	FIG038611	19003.88	4.93	179
A9ACU4 (GCSH_BURM1)	"Glycine cleavage system H protein OS=*Burkholderia multivorans* (strain ATCC 17616 / 249) OX=395019 GN=gcvH PE=3 SV=1"	70	2	*gcvH*	Unknown	E	FIG000296	13327.82	4.14	126
Biofilm – Band 5										
Q1BXV1 (ACP_BURCA)	"Acyl carrier protein OS=*Burkholderia cenocepacia* (strain AU 1054) OX=331271 GN=acpP PE=3 SV=1"	833	35	*acpP*	Cytoplasmic	IQ	FIG002328	8711.74	4.08	79
A0A160FL86	"RNA chaperone Hfq [*Burkholderia* sp. OLGA172]"	368	10	Bmul_1468 *hfq*	Cytoplasmic	R	FIG000583	8849.19	8.12	78
A0A1B4N3W2	"MULTISPECIES: CsbD family protein [*Burkholderia*]"	154	5		Unknown	S		7568.51	5.37	65
F0G2R9	"hypothetical protein B1M_12590 [*Burkholderia* sp. TJI49]"	141	2	Bmul_1385	Unknown	ND	FIG004899	11815.44	7.57	114
B5WDK9 UPI00018218D7	"nitrogen regulatory protein P-II [*Burkholderia* sp. H160]"	134	2	*glnB*	CytoplasmicMembrane	E	FIG006786	12241.18	5.87	112
B9AYU1	"hypothetical protein [*Burkholderia multivorans*]"	130	6	Bmul_3687	Unknown	SEGNOGG ND	FIG008837	8994.97	5.19	83
Q2SWG0	"thioredoxin [*Burkholderia thailandensis* E264]"	126	3	Bmul_1445	Cytoplasmic	OC		15721.97	8.46	146
UPI000B923569	"hypothetical protein CA831_04455, partial [*Burkholderia multivorans*]"	124	1	Bmul_4929	Unknown	S	FIG005605	12776.12	5.69	117
A9AJG1 (ATPD_BURM1)	"ATP synthase subunit delta OS=*Burkholderia multivorans* (strain ATCC 17616 / 249) OX=395019 GN=atpH PE=3 SV=1"	116	3	*atpH*	Cytoplasmic	C	FIG000262	19003.88	4.93	179
B1YRW3 (HIS2_BURA4)	"Phosphoribosyl-ATP pyrophosphatase OS=*Burkholderia ambifaria* (strain MC40-6) OX=398577	114	1	*hisE*	Unknown	E	FIG000871	13273.97	5.08	121

	GN=hisE PE=3 SV=1"									
A0A1J9PMB8	"histidine triad nucleotide-binding protein [*Burkholderia catarinensis*]"	110	3	Bmul_0338	Cytoplasmic	G F R		13046.95	6.35	121
A9ADI4 (RL7_BURM1)	"50S ribosomal protein L7/L12 OS=*Burkholderia multivorans* (strain ATCC 17616 / 249) OX=395019 GN=rplL PE=3 SV=1"	104	3	*rplL*	Unknown	J	FIG000141	12529.47	4.90	124
A0A1J9NKZ1	"hypothetical protein BFF94_09880 [*Burkholderia catarinensis*]"	102	2	Bmul_5227	Unknown	S	FIG021268	11497.01	5.44	110
A0A1J9PIU8	"cytochrome C [*Burkholderia catarinensis*]"	98	1	Bmul_0946	Periplasmic	C	FIG143085	12672.44	9.08	119
UPI0000E9AEDA	"Endoribonuclease L-PSP [*Burkholderia multivorans* ATCC 17616]"	98	2	Bmul_1781	Cytoplasmic	J	FIG012912	15339.34	6.03	140
A0A0E1SBK0	"conserved hypothetical protein [*Burkholderia pseudomallei* 305]"	96	1	Bmul_0436	Unknown	S	FIG005902	13342.29	9.72	119
A2W724	"hypothetical protein BDAG_00461 [*Burkholderia dolosa* AU0158]"	96	2	Bmul_0386	Unknown	J	FIG006243	18085.49	6.29	167
A0A1J9PLV5	"transporter [*Burkholderia catarinensis*]"	85	1	*osmY*	Periplasmic	R	FIG030456	11866.82	9.80	116
A9AJW4 (RL9_BURM1)	50S ribosomal protein L9 OS=*Burkholderia multivorans* (strain ATCC 17616 / 249) OX=395019 GN=rplI PE=3 SV=1	81	1	*rplL*	Cytoplasmic	J	FIG000205	16274.76	6.85	150
A0A0H3KES7	"antibiotic biosynthesis monooxygenase [*Burkholderia stabilis*]"	78	1	Bmul_1768	Cytoplasmic	S	FIG006866	10841.44	5.55	96
A0A1J9PT95	"hypothetical protein BFF94_26245 [*Burkholderia catarinensis*]"	71	1		Extracellular	S	FIG009175	18367.54	6.65	167
A0A0H3KGW6	"MULTISPECIES: cytochrome c [*Burkholderia*]"	71	2	Bmul_0947	Periplasmic	C		12814.71	8.60	119
F0FYS6	"CutA1 divalent ion tolerance protein [*Burkholderia* sp. TJI49]"	70	1	*cutA*	Unknown	P	FIG000880	11881.66	5.44	108
A0A0B1XUE8	"DNA-binding protein Bv3F [*Burkholderia cepacia*]"	69	1	Bmul_0190	Unknown	R	FIG005347	10811.29	9.82	97

[a] Uniprot accession number of each of the identified proteins by LC-MS/MS. [b] Score > 68 indicate identity or extensive homology ($p < 0.05$). [c] #matches: number of matching peptides during identification. [d] Prediction of the subcellular localization for each of the proteins according to Psortdb v3.0. [e] cluster of orthologous group (COG), functional categories for the identified proteins each of one is represented by one letters: M: Cell wall structure, biogenesis and outer membrane; N: cell motility; L: DNA replication, recombination and repair; K: transcription; J: translation including ribosomes biogenesis; O: posttranslational modifications; P: transport of inorganic ions; H: metabolism and transport of coenzymes; G: metabolism and transport of carbohydrates; C: production and conversion of energy; E: metabolism and transport of amino acids; Q: metabolism and transport of nucleotides; T: mechanisms of signal transduction; I: lipidic metabolism; F: metabolism and transport of nucleotides; R: general prediction function only; S: function unknown. [f] FIGFams, classification of proteins in according to the similarity along their full length and predicted to have the same function.

Table S2. List of proteins identified with LC-MS/MS in the outer membrane vesicles (OMVs) of *Burkholderia multivorans* C1576 after 7 days of grow at 30 °C.

Accession Number[a]	Description	Score[b]	# matches[c]	Gen name	Subcellular localization[d]	COG[e]	FIGFams[f]	MM (Da)	pI	Length (aa)
OMVs – Band 1										
UPI0006C79FF8	"MULTISPECIES: porin [*Burkholderia*]"	798	30	*opcP*	Outer Membrane	M	FIG005925	38840.78	9.44	373
A0A1B4NQ86	"MULTISPECIES: membrane protein [*Burkholderia*]"	446	17	Bmul_2265	Outer Membrane	M	FIG003465	23981.38	9.45	222
A0A1B4TUZ2	"MULTISPECIES: chaperonin GroEL [*Burkholderia*]"	297	16	Bmul_2528 *groL*	Cytoplasmic	O	FIG000056	57089.49	5.13	546
A0A0H3KF94	"iron complex outer membrane receptor protein [*Burkholderia multivorans* ATCC 17616]"	259	12	Bmul_1594	Outer Membrane	P	FIG138347	80489.79	7.02	739
UPI0004F8CCCF	"TonB-dependent hemoglobin/transferrin/lactoferrin family receptor [*Burkholderia multivorans*]"	221	7	Bmul_3338	Outer Membrane	P	FIG001878	81760.12	9.25	755
J4SPS8	"gram-negative porin [*Burkholderia multivorans* CF2]"	141	2	Bmul_4327	Outer Membrane	M	FIG021612	45839.57	9.27	439
B9C036	"adenosylhomocysteinase [*Burkholderia multivorans* CGD2]"	126	5	*ahcY*	Cytoplasmic	H	FIG000858	52235.44	5.78	472
A9AGW2	"Enolase OS=*Burkholderia multivorans* (strain ATCC 17616 / 249) OX=395019 GN=eno PE=3 SV=1"	114	4	*eno*	Cytoplasmic	G	FIG000118	45732.66	4.76	427
A0A1J9PMN7	"citrate (Si)-synthase [*Burkholderia catarinensis*]"	113	3	Bmul_4643	Cytoplasmic	C	FIG138183	48464.70	6.32	433
B9BYU4	"MULTISPECIES: aspartate/tyrosine/aromatic aminotransferase [*Burkholderia*]"	106	3	*aspC*	Cytoplasmic	E	FIG133263	42552.49	6.25	399
A0B2Z3	"5-methyltetrahydropteroyltriglutamate--homocysteine methyltransferase [*Burkholderia stabilis*]"	105	1	*metE*	Cytoplasmic	E	FIG000683	85543.33	5.52	777
UPI0006C7D81D	"aldehyde dehydrogenase family protein [*Burkholderia multivorans*]"	105	4	Bmul_3451	Cytoplasmic	C		53822.70	6.11	503
Q1BSN9	"S-adenosylmethionine synthase OS=*Burkholderia cenocepacia* (strain AU 1054) OX=331271 GN=metK PE=3 SV=1"	94	1	*metK*	Cytoplasmic	H	FIG000329	42708.36	5.04	395
UPI0000E8FDA0	conserved hypothetical protein [*Burkholderia multivorans* ATCC 17616]" Single-stranded DNA-binding protein	90	3	Bmul_5159	Unknown	S	FIG140295	41844.21	6.13	376
A0A2N9LBA0	"4-hydroxyphenylpyruvate dioxygenase [Burkholderiales bacterium]"	85	1	Bmul_0223 *hpd*	Cytoplasmic	E R	FIG001109	41485.10	5.87	368
B9BJR6	"outer membrane porin [*Burkholderia multivorans* CGD2]"	82	1	Bmul_4600	Outer Membrane	M	FIG005580	37356.11	6.96	360
A0A1J9PJ01	"leucine ABC transporter subunit substrate-binding protein LivK [*Burkholderia catarinensis*]"	80	1	Bmul_3623	Periplasmic	E		38988.78	8.88	372
UPI0006D6B49B	"TonB-dependent receptor [*Burkholderia multivorans*]"	75	1	Bmul_4173	Outer Membrane	P	FIG138304	76554.68	8.89	709
A0A1J9NHV6	"fumarylacetoacetase [*Burkholderia catarinensis*]"	74	1	*fah*	Cytoplasmic	Q	FIG001596	46931.09	5.45	434
A9ACU3	"Glycine dehydrogenase (decarboxylating) OS=*Burkholderia multivorans* (strain ATCC 17616 / 249) OX=395019 GN=gcvP PE=3 SV=1"	70	2	*gcvP*	Cytoplasmic	E	FIG000700	104328.6 1	5.99	975
OMVs – Band 2										

UPI0006C79FF8	"MULTISPECIES: porin [*Burkholderia*]"	583	26	*opcP*	Outer Membrane	M	FIG005925	38840.78	9.44	373
A0A104A8T0	"MULTISPECIES: porin [*Burkholderia cepacia* complex]"	320	15	*opcP*	Outer Membrane	M	FIG005925	38812.73	9.54	373
A0A1J9NN42	"hypothetical protein BFF94_00930 [*Burkholderia catarinensis*]"	292	9	Bmul_2265	Outer Membrane	M	FIG003465	24053.44	9.35	223
B9BD29	glutamate/aspartate ABC transporter periplasmic glutamate/aspartate-binding protein [*Burkholderia multivorans* CGD1]"	264	10	Bmul_2714 *gltI*		E T	FIG001171	41743.59	9.49	378
B9BXC6	"iron ABC transporter substrate-binding protein [*Burkholderia multivorans*]"	162	3	Bmul_2093	Periplasmic	P	FIG147656	37475.93	8.97	347
B4EFB0	Malate dehydrogenase OS=*Burkholderia cenocepacia* (strain ATCC BAA-245 / DSM 16553 / LMG 16656 / NCTC 13227 / J2315 / CF5610) OX=216591 GN=mdh PE=3 SV=1"	156	4	*mdh*	Unknown	C	FIG000455	35117.34	5.70	328
UPI0008639508	"beta-ketoacyl-ACP reductase [*Burkholderia contaminans*]"	151	1	Bmul_2181	Cytoplasmic	I Q R	FIG004337	25639.38	7.86	249
Q1BHJ3	"2,3,4,5-tetrahydropyridine-2,6-dicarboxylate N-succinyltransferase OS=*Burkholderia cenocepacia* (strain AU 1054) OX=331271 GN=dapD PE=3 SV=1"	117	1	*dapD*	Cytoplasmic	E	FIG000908	29479.56	5.66	275
A0A063B4P8	"aspartate/tyrosine/aromatic aminotransferase [*Burkholderia* sp. lig30]"	114	4	*aspC*	Cytoplasmic	E	FIG133263	42789.79	6.55	399
J4SJH5	"bacteriocin [*Burkholderia multivorans*]" (linocin M18)	103	2	Bmul_5413 *lin*	Cytoplasmic	S	FIG014104	28973.57	4.95	271
UPI000B924AC A	"ABC transporter substrate-binding protein partial [*Burkholderia multivorans*]"	95	3	Bmul_3347	Periplasmic	E T	FIG084786	24949.42	7.79	229
UPI0006C7D81D	aldehyde dehydrogenase family protein [*Burkholderia multivorans*]"	93	3	Bmul_3451	Cytoplasmic	C		53822.70	6.11	503
A0A1J9PV38	hypothetical protein BFF94_24240 [*Burkholderia catarinensis*]"	91	1	Bmul_0835	Periplasmic	S	FIG005833	25616.00	9.33	246
A0A0D5LAW2	"MULTISPECIES: ketol-acid reductoisomerase [*Burkholderia*]"	81	2	*ilvC*	Cytoplasmic	E H	FIG000244	36281.35	5.89	338
B9AZZ4	"aryl-alcohol dehydrogenase [*Burkholderia multivorans* CGD1]"	73	1	Bmul_4129	Cytoplasmic	C	FIG019686	39634.17	8.28	356
OMVs – Band 3										
J5C5K0	"MULTISPECIES: hypothetical protein [Proteobacteria]"	241	7	Bmul_1389	Unknown	S	FIG004850	19848.75	9.76	195
A0A3N8RJG5	"beta-ketoacyl-ACP reductase [*Burkholderia contaminans*]"	185	3	Bmul_2181	Cytoplasmic	I Q R	FIG004337	25639.38	7.86	249
B9BEK8	"ATP-dependent Clp protease proteolytic subunit ClpP [*Burkholderia multivorans* CGD1]"	124	3	Bmul_1349 *clpP*	Cytoplasmic	O U	FIG000028	22730.02	5.43	208
UPI000055A384	"redoxin family protein [*Burkholderia mallei*]"	119	3	Bmul_1331	Cytoplasmic	O	FIG000710	18916.31	5.02	169
UPI000B91EE63	"hypothetical protein CA830_37630 partial	111	3	Bmul_0858	Outer Membrane	M	FIG006619	16418.22	9.86	156

	[*Burkholderia multivorans*]"									
A0A238HB37	"Thiol peroxidase Tpx-type [*Burkholderia singularis*]"	108	1	*tpx*	Periplasmic	O	FIG002857	17057.38	5.35	164
A0A0E1UE91	"bacterioferritin [*Burkholderia mallei* NCTC 10247]"	104	2	Bmul_1081 *bfr*	Cytoplasmic	P	FIG001887	21020.87	4.80	180
A0A0H3KPT7	"phasin protein [*Burkholderia stabilis*]"	101	3	Bmul_1136	Unknown	N T	FIG005285	19741.35	5.96	188
B9B7H7	"hypothetical protein [*Burkholderia multivorans*]"	96	1	Bmul_4653	Unknown	S	FIG005607	17625.45	9.76	167
B9BCY0	"YceI family protein [*Burkholderia multivorans* CGD1]"	89	1	Bmul_2669	Unknown	S	FIG005976	20383.08	6.29	188
UPI000B924C65	"riboflavin synthase partial [*Burkholderia multivorans*]"	84	1	*ribE*	Cytoplasmic	H	FIG000273	8243.37	4.27	82
B9B376	"MULTISPECIES: thiol:disulfide interchange protein DsbA/DsbL [*Burkholderia*]"	82	1	*dsbA*	Periplasmic	O C	FIG133105	23276.92	9.05	212
A0A1J9NNJ0	hypothetical protein BFF94_09135 [*Burkholderia catarinensis*]"	82	4	Bmul_1507	Outer Membrane	M	FIG005063	22536.02	8.67	213
Q0BH97	"conserved hypothetical protein [*Burkholderia ambifaria* AMMD]"	73	2	Bmul_2263	Unknown	S	FIG005158	21364.60	9.36	200
B9B2C3	"hypothetical protein [*Burkholderia multivorans*]"	72	1	Bmul_3407	Cytoplasmic		FIG028923	20407.30	9.83	184
F0GBW7	"superoxide dismutase [*Burkholderia* sp. TJI49]"	72	1	*sod*	Periplasmic	P	FIG133139	15721.57	5.64	147
OMVs – Band 4										
UPI000B923569	"hypothetical protein CA831_04455 partial [*Burkholderia multivorans*]"	120	2	Bmul_4929	Unknown	S	FIG005605	12776.12	5.69	117
Q1BXV1	Acyl carrier protein OS=*Burkholderia cenocepacia* (strain AU 1054) OX=331271 GN=acpP PE=3 SV=1"	120	2	*acpP*	Cytoplasmic	I Q	FIG002328	8711.74	4.08	79
Q2SWG0	thioredoxin [*Burkholderia thailandensis* E264]"	96	2	Bmul_1445	Cytoplasmic	O C		15721.97	8.46	146
A0A0E1SBK0	"conserved hypothetical protein [*Burkholderia pseudomallei* 305]"	89	1	Bmul_0436	Unknown	S	FIG005902	13342.29	9.72	119
J4RCD6	"MULTISPECIES: RidA family protein [*Burkholderia*]"	76	2	Bmul_0927	Unknown	J		15740.07	5.91	152
Q6H8I8	"outer membrane lipoprotein [*Burkholderia multivorans*]"	83	1	Bmul_2785 *slyB*	Outer Membrane	M	FIG005726	15456.55	9.76	157
A9ADI4	"50S ribosomal protein L7/L12 OS=*Burkholderia multivorans* (strain ATCC 17616 / 249) OX=395019 GN=rplL PE=3 SV=1"	77	1	*rplL*	Unknown	J	FIG000141	12529.47	4.90	124
P0DMK4	"DNA-binding protein HU-alpha OS=*Burkholderia pseudomallei* (strain K96243) OX=272560 GN=hupA PE=3 SV=1"	73	1	*hupB hupA?*	Cytoplasmic	L	FIG023522	9621.08	10.2 0	92
OMVs – Band 5										
Q1BXV1	"Acyl carrier protein OS=*Burkholderia cenocepacia* (strain AU 1054) OX=331271 GN=acpP PE=3 SV=1"	148	4	*acpP*	Cytoplasmic	I Q	FIG002328	8711.74	4.08	79
UPI000B923569	"hypothetical protein CA831_04455 partial [*Burkholderia multivorans*]"	130	2	Bmul_4929	Unknown	S	FIG005605	12776.12	5.69	117
A0A0E1SBK0	"conserved hypothetical protein [*Burkholderia*	118	2	Bmul_0436	Unknown	S	FIG005902	13342.29	9.72	119

	pseudomallei 305]"									
P0DMK4	"DNA-binding protein HU-alpha OS=*Burkholderia pseudomallei* (strain K96243) OX=272560 GN=hupA PE=3 SV=1"	86	2	*hupB hupA?*	Cytoplasmic	L	FIG023522	9621.08	10.20	92
UPI00015FD84E	"conserved hypothetical protein [*Burkholderia multivorans* ATCC 17616]"	77	1	Bmul_4062	Unknown	NI	FIG033112	16839.15	10.20	156

[a] Uniprot accession number of each of the identified proteins by LC-MS/MS. [b] Score > 68 indicate identity or extensive homology ($p < 0.05$). [c] #matches: number of matching peptides during identification. [d] Prediction of the subcellular localization for each of the proteins according to Psortdb v3.0. [e] cluster of orthologous group (COG), functional categories for the identified proteins each of one is represented by one letters: M: Cell wall structure, biogenesis and outer membrane; N: cell motility; L: DNA replication, recombination and repair; K: transcription; J: translation including ribosomes biogenesis; O: posttranslational modifications; P: transport of inorganic ions; H: metabolism and transport of coenzymes; G: metabolism and transport of carbohydrates; C: production and conversion of energy; E: metabolism and transport of amino acids; Q: metabolism and transport of nucleotides; T: mechanisms of signal transduction; I: lipidic metabolism; F: metabolism and transport of nucleotides; R: general prediction function only; S: function unknown. [f] FIGFams, classification of proteins in according to the similarity along their full length and predicted to have the same function.

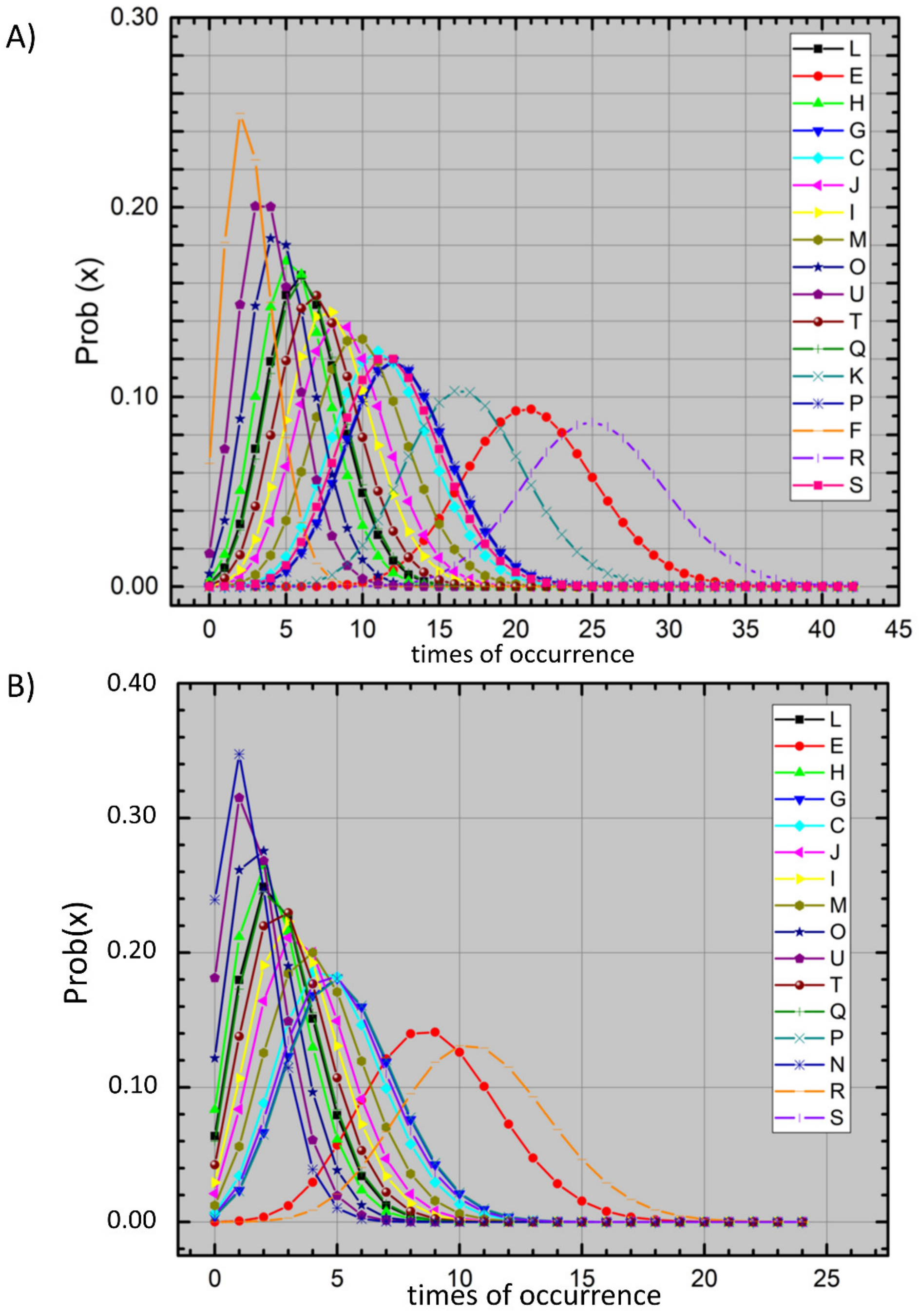

Figure S1. Hypergeometric distribution of the probabilities Prob(x) of finding proteins from a determine cluster of orthologous group (COG) category a certain number of times "x" of (**A**) Biofilm and (**B**) outer membrane vesicles (OMVs).

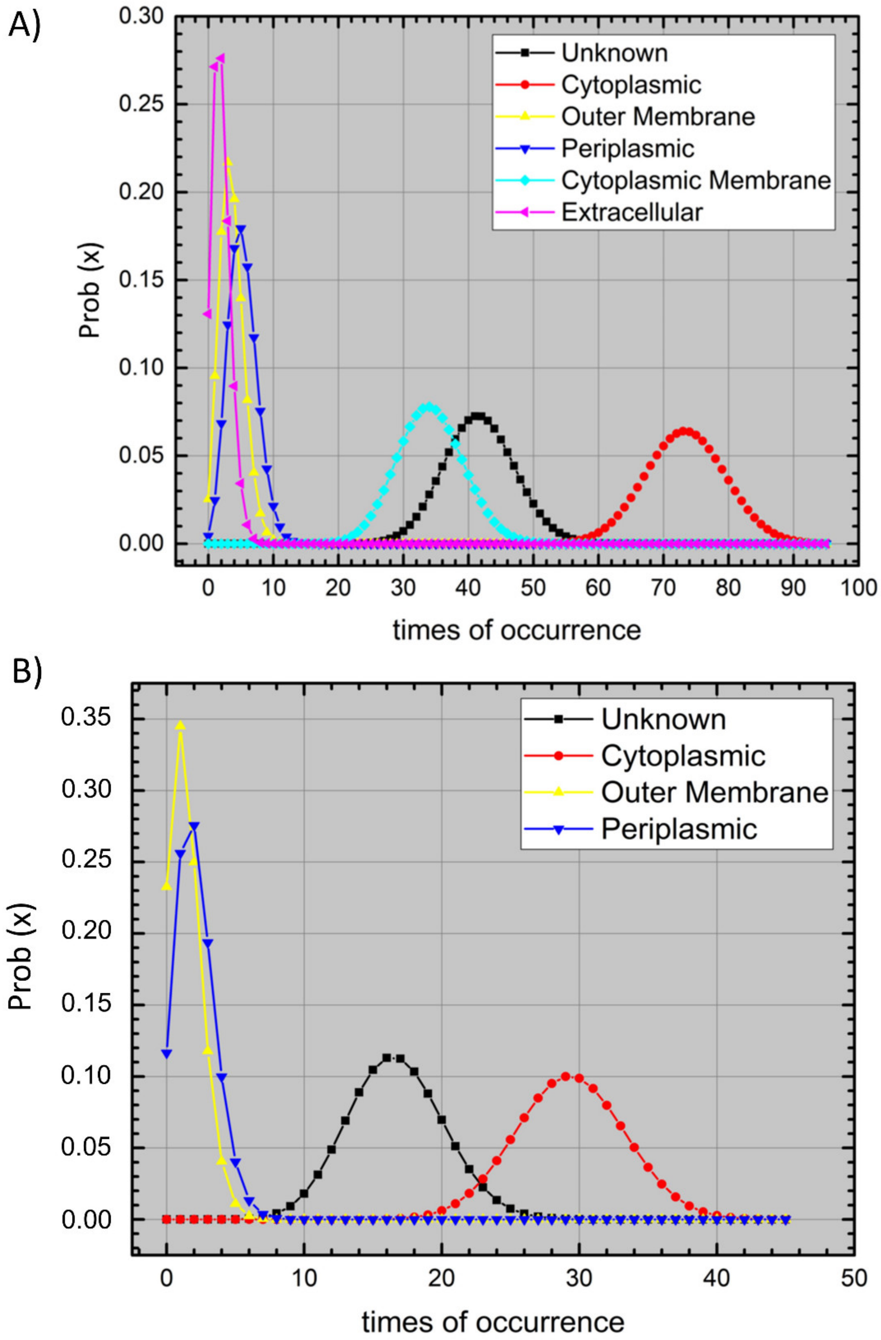

Figure S2. Hypergeometric distribution of the probabilities Prob(x) of finding proteins from a determine subcellular localization a certain number of times "x" of (**A**) Biofilm and (**B**) outer membrane vesicles (OMVs).
